# Congenital Versus Isolated Traumatic Radial Head Dislocation in Adults: A Diagnostic Dilemma With an Algorithmic Approach

**DOI:** 10.7759/cureus.90880

**Published:** 2025-08-24

**Authors:** Konstantinos Zampetakis, Constantinos Chaniotakis, Petros Kapsetakis, Alexandros Tsioupros, Ioannis M Stavrakakis

**Affiliations:** 1 Department of Orthopaedics and Traumatology, "Venizeleio" General Hospital of Heraklion, Heraklion, Crete, GRC

**Keywords:** congenital radial head dislocation, diagnostic algorithm, differential diagnosis, elbow instability, radial head dysplasia, traumatic radial head dislocation

## Abstract

Congenital radial head dislocation (CRHD) is a rare elbow condition that often remains undiagnosed until adulthood due to its asymptomatic nature. In contrast, isolated traumatic radial head dislocation (TRHD) is another uncommon entity that results from an elbow injury. Given their rarity and overlapping clinical features, distinguishing CRHD from isolated TRHD, particularly in adults with a history of trauma, poses a significant diagnostic dilemma. This review aims to enhance awareness about both conditions, and it is the first to address an in-depth comparison. A novel dual-algorithm approach is proposed as well to overcome the diagnostic challenges and guide clinicians’ decisions. PubMed and Scopus were searched to identify reported cases of CRHD and isolated TRHD in adults. Key aspects, including epidemiology, clinical presentation, diagnostic modalities, and treatment strategies, were analyzed. Two diagnostic algorithms were developed based on clinical and radiographic parameters.

CRHD is often asymptomatic until adulthood, typically lacks a history of trauma, shows bilateral involvement, and is frequently associated with congenital abnormalities. Conversely, isolated TRHD presents acutely with pain and a restricted range of motion (ROM) post-trauma. Imaging findings are crucial to differentiate the former two entities. Treatment differs significantly; CRHD is typically managed conservatively unless symptomatic, while isolated TRHD often requires closed reduction, with surgery reserved for irreducible cases or instability. CRHD and isolated TRHD in adults are exceptionally rare, and differentiation is crucial for proper management. This review highlights the diagnostic challenges of CRHD first identified in adulthood and emphasizes the value of clinical suspicion, imaging, and individualized treatment planning. Finally, the dual-algorithm approach provides the first structured diagnostic model to assist differential diagnosis of CRHD and isolated TRHD in adults, offering a cost-effective pathway and a more comprehensive option when initial evaluation is inconclusive.

## Introduction and background

Congenital radial head dislocation (CRHD) is a rare clinical entity with an incidence ranging from 0.06% to 0.16%, although it remains the most common congenital anomaly of the elbow [1-5]. It can manifest as part of a broader systemic syndrome or hereditary condition, in up to 60% of cases, suggesting multifactorial insults to embryologic elbow development [1,6-8]. However, the exact etiology of CRHD remains unknown and has not been investigated in depth [3,7]. In some cases, injuries such as "pulled elbow" can occur during birth or early life and cause dislocation of the radial head due to laxity of the annular ligament, which encircles the radial head. If the dislocation remains unreduced, it can present with analogous deformity of CRHD [3]. Clinical symptoms in childhood are usually absent or minor, and this extends until adolescence, hence conservative treatment with regular follow-up remains the standard approach [2,3,9,10]. Surgical treatment is indicated for cases with persistent pain, loss of range of motion (ROM), and elbow joint instability [1,10].

Diagnosing CRHD later in adulthood can be challenging, and it is an exceptionally rare phenomenon. According to Gao et al’s literature review in 2023, only nine cases of CRHD in adult patients have been described [2]. CRHD is frequently identified incidentally during imaging studies performed after trauma [11]. Therefore, the presence of a traumatic event in relevant history can make the identification of CRHD even harder, as it brings up isolated traumatic radial head dislocation (TRHD) in the differential diagnosis [12]. Typically, radial head dislocations are associated with more complex injuries, including Monteggia fractures, which are defined as fractures of the proximal ulna with concurrent dislocation of the radial head [13-15]. On the other hand, isolated TRHD constitutes another uncommon condition in adulthood, with fewer than 40 cases being reported in the orthopaedic literature [13-18]. Distinguishing CRHD from isolated TRHD in the setting of elbow trauma is of paramount importance, especially in unilateral cases, in order to avoid long-term complications, such as elbow joint stiffness and instability, osteoarthritis, and neuropathies [1,4,12,14,19].

This study is a narrative review presenting CRHD and isolated TRHD based on the existing knowledge. This is the first review addressing an in-depth comparison of the two clinical entities and highlighting the diagnostic complexities of CRHD when first recognized in adulthood. By conducting a side-by-side evaluation of CRHD and isolated TRHD, the present study aims to enhance awareness about both conditions and help clinicians manage this clinical dilemma by making more informative diagnostic and therapeutic decisions. Additionally, this review introduces a dual-algorithm diagnostic model, the first of its kind in the literature, designed to provide a practical framework for differentiating CRHD from isolated TRHD in adults.

## Review

Methods

The PubMed and Scopus databases were searched for articles on CRHD and/or isolated TRHD, using the terms "congenital radial head dislocation" and "isolated traumatic radial head dislocation" combined with "adults". The search covered the period from January 2000 to August 2025, and only articles published in English were included. In addition, a backward citation search of relevant articles was conducted to ensure comprehensive coverage of the available literature. Main characteristics, including epidemiology, clinical image, imaging findings, and treatment strategies, were documented and analyzed. Given the extreme rarity of CRHD and isolated TRHD in adults and the heterogeneity of available reports, a quantitative synthesis was not feasible; therefore, a narrative synthesis approach was applied. Based on these findings, two complementary diagnostic algorithms were constructed: one offering a cost-effective initial approach suitable for most clinical environments, and another for cases where radiographs are inconclusive or clinical suspicion remains high.

Results

A total of nine cases of CRHD and 22 cases of isolated TRHD were found in the existing literature. An overview of epidemiology, clinical presentation, imaging findings, treatment options, and diagnostic algorithms is presented below, highlighting the key differences.

Epidemiology

The incidence of CRHD is 6-16 new cases per 10.000 patients [2,4]. However, the incidence of CRHD in adults is believed to be much lower, as only nine such cases have been previously documented in the literature [2]. While most cases of isolated TRHD are reported in children, adult presentations, which are the exclusive focus of this review, remain less frequently described [15]. Isolated TRHD in adults is a very uncommon injury, and even though there is no bibliographic data about the accurate incidence, it is believed to be exceptionally low, knowing that fewer than 40 cases have been described [13-18]. In CRHD, the radial head dislocates posteriorly in 65% of cases, followed by anterior and lateral dislocations, accounting for about 15% each [1-3,7,9,20]. The same applies to isolated TRHD. The most prevalent type of dislocation is posterior, but there are also reports of anteromedial and anterior dislocations [14,15,18,21,22].

Clinical Presentation

There is a large spectrum of clinical manifestations regarding CRHD. This condition is frequently missed during birth and early childhood, as it is asymptomatic or has minor symptoms. Patients with CRHD usually develop symptoms in adolescence [1-4,9,23]. Normally, there is no history of trauma, and the symptoms involve mainly elbow pain and instability, snapping, valgus deformity, ROM limitations, and neuropathy, such as carpal tunnel syndrome [1,2,4,23]. Interestingly, posterior interosseous nerve (PIN) palsy can progressively occur as a sole symptom, due to impingement along the Arcade of Frohse [4]. Median nerve compression has also been described in an untreated case of CRHD [24]. The possible reason why CRHD in adults is diagnosed much less commonly is that such adults typically have only mild symptoms and no relevant functional impairment, probably because of the compensatory motion of the wrist and shoulder, even though minor limitations can occasionally be present [2,4].

On the contrary, patients with isolated TRHD present with a relevant history of trauma, including various injury mechanisms. Most authors refer to indirect injury mechanisms, such as hyperpronation forces to an already pronated forearm or a fall in a hyperextended and supinated elbow position [14-17,21]. Isolated TRHD can present with elbow swelling, tenderness, ecchymosis, and limitations in ROM [14-16,18,21,22]. Most typically, in isolated TRHD, there is complete loss of pronation and supination but preservation of elbow flexion and extension, sometimes leading to late presentation or misdiagnosis [13,15,16]. However, there is a report of an atypical case with no restrictions in pronosupination, but limitations in flexion and extension [21].

A significant difference between the two clinical entities is that CRHD typically presents with bilateral involvement [1,7,9,11]. There are several reports that it can occur either bilaterally or unilaterally, but bilateral radial head dislocations are characteristic of a congenital disorder [1-3,7,9,23]. In unilateral cases, the difficulty in diagnosis increases dramatically [18]. Moreover, unlike isolated TRHD, CRHD is often associated with other systemic conditions, such as nail-patella, Klippel-Feil, and Steinfeld syndromes, lower limb abnormalities, scoliosis, longitudinal radial insufficiency, intellectual disability, hereditary multiple exostoses, Apert syndrome, ulnar dysplasia, osteogenesis imperfecta, and trisomy 8 and 12 [1-4,6,9,25,26].

Imaging Findings

Imaging studies are also crucial in the differential diagnosis. Plain X-ray films are broadly available, inexpensive, and should be the first-line radiologic examination of the painful elbow [27]. McFarland first described the following radiographic criteria of CRHD: (a) increased length ratio of radius relative to ulna, (b) absent or hypoplastic capitellum, (c) grooving of the distal radius, (d) prominent ulnar epicondyle, (e) partially defective trochlea and (f) dome-shaped radial head with a long narrow neck [1,3,9,28]. Further clinical criteria were later proposed by Mardam-Bey and Ger, including: (a) bilateral involvement, (b) associated congenital conditions, (c) familial occurrence, (d) absent history of trauma, (e) failed closed reduction maneuver, and (f) dislocation present at birth [2-4,9,29].

When concomitant fractures are present, the diagnosis of CRHD can be much more difficult [19]. Arthrography, using X-rays or computed tomography (CT), can be a valuable examination as it assesses if the position of the radial head is intracapsular, suggesting CRHD, or extracapsular, which directs the diagnosis towards isolated TRHD [12]. CT is also important for excluding concurrent fractures and for confirming the dislocated position of the radial head, as isolated dislocations may be easily missed in simple X-rays [14]. Furthermore, magnetic resonance imaging (MRI) can help with identifying bone marrow edema and possible cartilaginous or ligamentous injuries, favoring isolated TRHD, as radial head dislocation is not feasible without the rupture of the annular ligament [12,16,21,22]. Finally, both CT and MRI may reveal subtle deformities of the radial head and capitellum that can be missed on standard radiographs [9,30].

Additional Diagnostic Considerations and Mimicking Conditions

Although the main focus of this review is the differentiation between CRHD and isolated TRHD in the adult population, several other mimicking conditions may present with overlapping features and obscure the diagnostic process [3,31]. Neglected Monteggia lesions, which are characterized by proximal ulna malunion with concurrent radial head dislocation, can be diagnosed in adults for the first time if the primary injury was overlooked during childhood. The prolonged radial head dislocation can lead to several deformities developing at the humeroradial joint, resembling a CRHD clinically or radiologically [32,33]. Proximal radioulnar synostosis, either congenital or post-traumatic, can cause ROM limitations in pronation and supination and may also include the radial head. A careful patient history about previous elbow injuries and scrutiny of imaging studies are essential in these cases [34-36]. Moreover, premature ossification of the physeal plates of the lateral humeral condyle or radial head can cause deformities such as angular deformities and changes in the relative bone length ratio, which may distort the normal anatomy of the elbow joint. This alteration may be attributed to a previous elbow trauma and can lead to abnormal positioning of the radial head that mimics or is misdiagnosed as radial head dislocation on clinical and radiographic evaluation [3,37]. Despite the available diagnostic tools, prompt identification of these potential mimickers is critical to avoid misinterpretations and guide appropriate management.

Treatment Options

Concerning CRHD, there is no established consensus about the most appropriate treatment option in adulthood, and usually the management is individualized, depending on the symptoms [2,4]. The variability in clinical presentation can make treatment choice demanding [23,38]. Conservative treatment with close observation and activity modifications is the gold-standard treatment when patients are asymptomatic or have only mild symptoms [2,9,23]. Closed reduction is amenable only in some cases of CRHD diagnosed in childhood and is usually unsuccessful in adults [2].

Operative treatment is reserved for patients with refractory symptoms or cosmetic concerns and should be chosen with caution [9,12,23]. Surgical treatment options in adults include radial head resection, rotational osteotomy of the radius, ulnar osteotomy, reconstruction of the annular ligament, and arthroscopic release of the annular ligament. Each choice has specific indications, as well as advantages and disadvantages [2,3,5,9,23,38]. Radial head resection is not recommended before skeletal maturity. It leads to significant pain relief and slight improvements in pronosupination, but it does not improve flexion and extension, and in the long-term run, resection can cause valgus deformity, instability of the elbow joint, and pain to the distal radioulnar joint [2,3,23,38]. Rotational osteotomy of the radius takes advantage of the normal functional anatomy of the elbow and reduces biceps brachii tension, which is considered a principal risk factor for recurrent anterior dislocations [2]. Even with the addition of radial osteotomy, there is a high likelihood of instability following open reduction because of the dome-shaped radial head, which is present in most cases. Hence, to avoid recurrence, radial osteotomy should be better considered in cases where radial head morphology is close to normal [39]. Proximal ulnar osteotomy can redirect the plane of the proximal radioulnar joint and has also been described as a surgical option for anterior CRHD cases. However, its use in the adult population should be weighed carefully, due to possible complications, such as supination weakness and limited functional improvement [2,39,40]. In the setting of PIN compression, surgical nerve exploration and neurolysis are an option when mechanical compression is considered, after a non-effective period of six weeks of conservative treatment [4].

Closed reduction is successful and has favorable outcomes for most cases of isolated TRHD, despite the lack of clear guidelines regarding management of this condition [14-16,18,21]. Most authors suggest a post-reduction immobilization period for six weeks in flexion and supination [16]. Unsuccessful closed reduction maneuvers in isolated TRHD can occur due to soft tissue interposition, such as the brachialis tendon, annular ligament, biceps tendon, and anterior joint capsule [18,22,41]. Chronic injuries or failed closed reduction should be treated with open reduction, including annular ligament reconstruction or radial head resection [13,14,16,18,21]. Instability may occur after a successful reduction. In such cases, radial head excision, K-wire fixation, and modified Bell-Tawse repair are indicated [18].

The key differences between CRHD and isolated TRHD are summarized in Table 1.

**Table 1:** Summary of the key differences between congenital and isolated traumatic radial head dislocation. ROM: range of motion

Parameters	Congenital Radial Head Dislocation	Isolated Traumatic Radial Head Dislocation
Incidence	Very rare (0.06-0.16%)	Very rare (exact incidence unknown)
Associated conditions	Very common (60%) (nail-patella syndrome, scoliosis, osteogenesis imperfecta, etc.)	-
Direction of dislocation	Posterior (65%)>Anterior (18%)> Lateral (17%)	Posterior > Anterior > Anteromedial
Bilateral involvement	Most cases	-
Clinical presentation	-No history of trauma -Mainly asymptomatic or mild symptoms, full ROM (in most cases) -Less commonly: Elbow pain, instability, snapping, limited ROM, neuropathies	-History of trauma -Elbow pain, swelling, tenderness, ecchymosis, limited ROM -Complete loss of pronation and supination -Preservation of flexion and extension
X-ray findings	-Dislocation of radial head -Bilateral involvement -Dome-shaped radial head -Hypoplastic or absent capitellum -Grooving of the distal radius -Increased radius to ulna length ratio -Partially defective trochlea -Prominent medial epicondyle	-Dislocation of radial head -Normal radial head and capitellum morphology
Arthrography	Radial head intracapsular	Radial head extracapsular
CT findings	-Similar to x-ray -Confirm dislocated positions of radial head -Exclude concurrent bony injuries	-Confirm dislocated positions of radial head -Exclude concurrent bony injuries
MRI findings	-Similar to x-ray	-Bone bruise -Ligamentous injury -Cartilaginous injury
Treatment	-Conservative is “gold-standard” for mild cases -Radial head resection -Rotational radial osteotomy -Ulnar osteotomy -Annular ligament reconstruction -Arthroscopy	-Closed reduction and immobilization -Open reduction -K-wire fixation -Annular ligament reconstruction -Radial head resection

Suggested diagnostic algorithm

The diagnostic dilemma of CRHD versus isolated TRHD in adults remains challenging, especially in a trauma setting, where clinical findings may overlap. The rarity of both conditions makes the prompt identification even harder, especially for younger colleagues. To facilitate clinical decision-making, we propose a diagnostic approach consisting of two complementary algorithms. To the best of our knowledge, this is the first algorithmic approach presented in the literature that evaluates the diagnosis of suspected CRHD and isolated TRHD in the adult population, following trauma.

**Figure 1:**
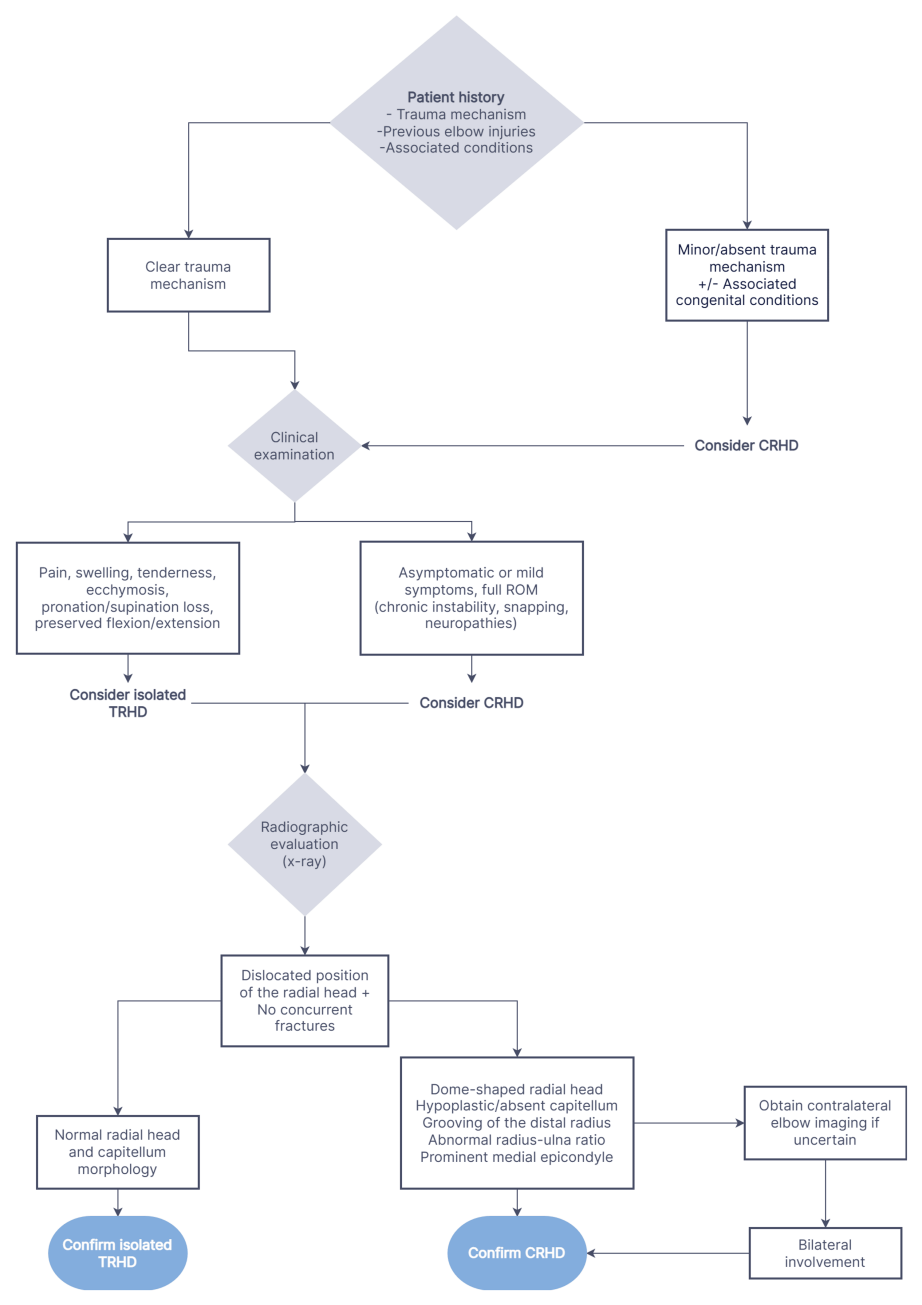
Primary diagnostic algorithm for suspected radial head dislocation in adults. CRHD: congenital radial head dislocation; TRHD: traumatic radial head dislocation

Figure 1 illustrates the primary diagnostic flowchart algorithm, which is constructed as a practical and inexpensive approach, applicable in most clinical settings, without the requirement of advanced imaging modalities.

The process begins with a detailed patient history, including the referred mechanism of injury, any previous elbow trauma, and medical conditions associated with CRHD. A thorough clinical examination is the next step of the approach, where acute findings, such as pain, swelling, tenderness, and limitation of forearm rotation, are evaluated. A distinct mechanism of injury in combination with these acute signs should prompt consideration of isolated TRHD. In contrast, a minor or absent trauma mechanism raises the suspicion of CRHD, especially in the context of other known congenital conditions.

Following the clinical examination, radiographic evaluation remains the keystone of primary assessment. Plain X-ray films are used to confirm the dislocated position of the radial head and to evaluate for characteristic features. Findings, including a dome-shaped radial head, hypoplastic or absent capitellum, abnormal ratio between radius and ulna, or a prominent medial epicondyle, are highly indicative of CRHD. On the other hand, a normal radial head and capitellum morphology in the setting of clear acute trauma aligns with a diagnosis of isolated TRHD. In ambiguous cases, contralateral elbow imaging may provide a valuable baseline reference.

**Figure 2:**
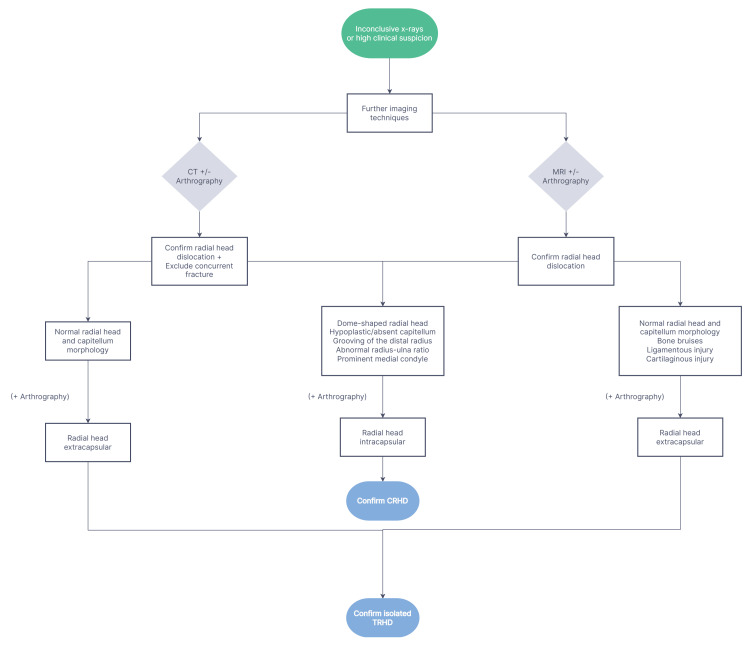
Complementary diagnostic algorithm for cases with inconclusive radiographs or persistent high clinical suspicion. CRHD: congenital radial head dislocation; TRHD: traumatic radial head dislocation

Figure 2 presents the secondary diagnostic algorithm, designed for situations where the first-line workup does not yield a definite diagnosis, either due to inconclusive X-rays or persistent high clinical suspicion.

In such cases, the use of advanced imaging techniques is recommended. CT and MRI can both aid the diagnostic procedure, and the choice between them rests with the physician’s clinical judgement and preference. However, a CT scan is generally preferred in the emergency setting, considering its wide availability and its ability to provide excellent bony detail and exclude any associated bony injury. MRI offers the advantage of detecting soft tissue pathologies, such as ligamentous injuries or cartilage lesions, as well as subtle bone bruises. It can be used when the results of the CT scan are inconclusive or in cases where soft tissue evaluation is the primary focus. The additional use of arthrography in complex cases provides valuable information by determining the extracapsular or intracapsular position of the radial head. Morphological anomalies of the radiocapitellar joint are suggestive of CRHD. On the contrary, a normal morphological appearance of the radial head and capitellum, along with evidence of acute trauma, can confirm the diagnosis of isolated TRHD.

## Conclusions

CRHD and isolated TRHD in adult life are exceptionally rare conditions, and their differential diagnosis can be puzzling, especially when there is a history of trauma. Through this comprehensive literature review, the direct comparison of CRHD and isolated TRHD highlights the diagnostic intricacies of delayed identification of CRHD in adults. By clarifying the differences in terms of epidemiology, clinical presentation, radiologic findings, and treatment options, this study raises orthopaedic surgeons’ awareness of both clinical entities and aids in clinical decision-making for their optimal management.

The proposed dual-algorithm approach offers a detailed framework for clinicians managing patients with suspected radial head dislocation. The primary algorithm is simple, cost-effective, and applicable in most clinical environments, whereas the secondary algorithm comprises a valuable clinical tool when initial evaluation is inconclusive. Combined, they represent the first diagnostic model proposed in the literature to assist physicians in distinguishing CRHD from isolated TRHD in adults. Future research with standardized outcome measures and long-term follow-up data for both conditions, although it may be difficult due to their rarity, is necessary to provide clearer guidance.
